# Nitric oxide and pH modulation in gynaecological cancer

**DOI:** 10.1111/jcmm.12921

**Published:** 2016-07-29

**Authors:** Carlos Sanhueza, Joaquín Araos, Luciano Naranjo, Eric Barros, Mario Subiabre, Fernando Toledo, Jaime Gutiérrez, Delia I. Chiarello, Fabián Pardo, Andrea Leiva, Luis Sobrevia

**Affiliations:** ^1^ Cellular and Molecular Physiology Laboratory (CMPL)Division of Obstetrics and GynaecologySchool of MedicineFaculty of MedicinePontificia Universidad Católica de ChileSantiagoChile; ^2^ Department of Basic SciencesFaculty of SciencesUniversidad del Bío‐BíoChillánChile; ^3^ Cellular Signalling and Differentiation Laboratory (CSDL)School of Medical TechnologyHealth Sciences FacultyUniversidad San SebastianSantiagoChile; ^4^ Department of PhysiologyFaculty of PharmacyUniversidad de SevillaSevilleSpain; ^5^ University of Queensland Centre for Clinical Research (UQCCR)Faculty of Medicine and Biomedical SciencesUniversity of QueenslandHerstonQLDAustralia

**Keywords:** nitric oxide, pH, NHE, ovarian cancer

## Abstract

Nitric oxide plays several roles in cellular physiology, including control of the vascular tone and defence against pathogen infection. Neuronal, inducible and endothelial nitric oxide synthase (NOS) isoforms synthesize nitric oxide. Cells generate acid and base equivalents, whose physiological intracellular concentrations are kept due to membrane transport systems, including Na^+^/H^+^ exchangers and Na^+^/HCO_3_^−^ transporters, thus maintaining a physiological pH at the intracellular (~7.0) and extracellular (~7.4) medium. In several pathologies, including cancer, cells are exposed to an extracellular acidic microenvironment, and the role for these membrane transport mechanisms in this phenomenon is likely. As altered NOS expression and activity is seen in cancer cells and because this gas promotes a glycolytic phenotype leading to extracellular acidosis in gynaecological cancer cells, a pro‐inflammatory microenvironment increasing inducible NOS expression in this cell type is feasible. However, whether abnormal control of intracellular and extracellular pH by cancer cells regards with their ability to synthesize or respond to nitric oxide is unknown. We, here, discuss a potential link between pH alterations, pH controlling membrane transport systems and NOS function. We propose a potential association between inducible NOS induction and Na^+^/H^+^ exchanger expression and activity in human ovary cancer. A potentiation between nitric oxide generation and the maintenance of a low extracellular pH (*i.e*. acidic) is proposed to establish a sequence of events in ovarian cancer cells, thus preserving a pro‐proliferative acidic tumour extracellular microenvironment. We suggest that pharmacological therapeutic targeting of Na^+^/H^+^ exchangers and inducible NOS may have benefits in human epithelial ovarian cancer.

## Introduction

Gynaecological cancers affect women mainly after fertile age 1. Ovarian cancer is a type of gynaecological cancer, and epithelial ovarian cancer accounts for ∼90% of all ovarian cancer types 2. Epithelial ovarian cancer could be type I or II. Type I epithelial ovarian cancer characterizes by a low‐grade serous, mucinous, endometrioid and clear‐cell histotype, and type II epithelial ovarian cancer corresponds to advanced serous, endometrioid or undifferentiated histotype 2, 3. Gynaecological cancers associate with altered synthesis of key molecules, such as the gas nitric oxide, involved in the modulation of cell proliferation and metabolism 4, 5. Nitric oxide shows a short half‐life in the blood (varying between 0.002 and 2 sec. depending on oxygen (O_2_) concentration) 6 and is key in vascular reactivity and blood coagulation 7, 8, 9.

The nitric oxide is a side product from the conversion of the semi‐essential cationic amino acid L‐arginine into L‐citrulline by at least three different nitric oxide synthases (NOS), *i.e*. neuronal NOS (NOS‐1 or nNOS), inducible NOS (iNOS or NOS‐2) and endothelial NOS (eNOS or NOS‐3) 10. Nitric oxide has a dual effect, acting as a tumour promoter or by preventing tumour growth 11. Since a change in the intracellular pH (pHi) modulates nitric oxide synthesis 12, 13, pHi modulatory mechanisms may play a role in nitric oxide synthesis. As (*i*) there is an evidence of lack of information and unclear mechanisms involved in human ovarian epithelial cancer, (*ii*) a coupling between nitric oxide signalling and cell metabolism in ovarian cancer has been proposed 14, 15, and (*iii*) membrane transport mechanisms directly involved in the modulation of pHi/extracellular pH (pHo) are expressed in human ovarian cancer cells 16, we focused this review on a potential connection between the mechanisms controlling pHi and the NOS activity in human gynaecological cancers.

## Plasma membrane mechanisms of pH control

**Figure 1:**
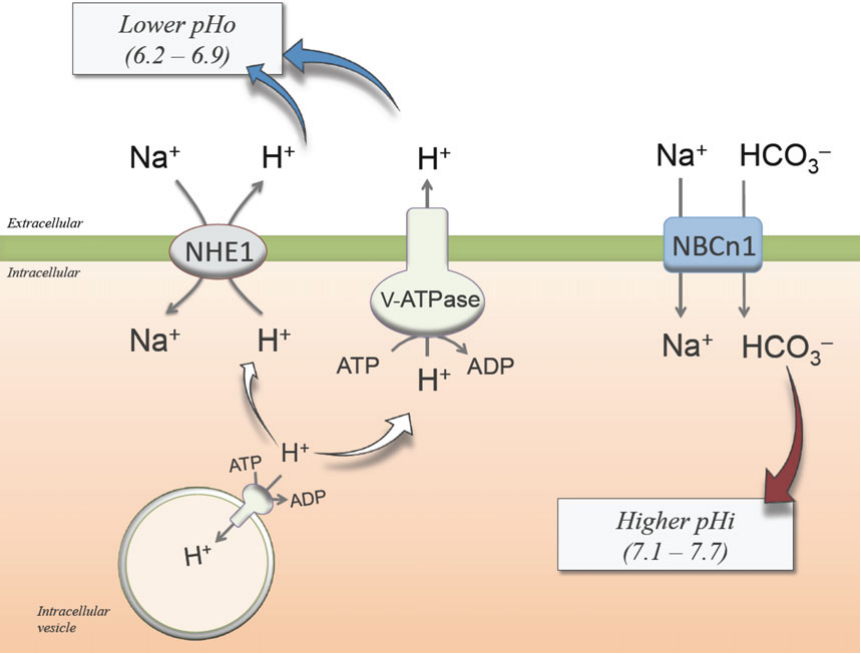
Membrane transport systems involved in the regulation of pH in gynaecological cancers. At least three different extracellular (pHo) and intracellular (pHi) pH regulatory systems are described in tissues and cells from human gynaecological cancers. The sodium (Na^+^), proton (H^+^) exchanger isoform 1 (NHE1) is a membrane transporter that extrudes one intracellular H^+^ in an exchange with one extracellular Na^+^. The vacuolar H^+^‐ATPases (V‐ATPase) located at the plasma membrane mediate active H^+^ efflux at expenses of ATP hydrolysis into ADP. In addition, the activity of V‐ATPases located at intracellular vesicles contributes to removing H^+^ from the cytoplasmic medium. The activity of NHE1 and V‐ATPases leads to (blue arrows) extracellular acidification (*lower pHo*). Coexpressed with these membrane transporters, the Na^+^/bicarbonate (HCO_3_^−^) electroneutral cotransporter 1 (NBCn1) takes up Na^+^ and HCO_3_^−^ from the extracellular media leading to (brown arrow) intracellular alkalization (*higher pHi*). NBCn1 activity contributes to extracellular acidification, and NHE1 and V‐ATPases activity contributes to intracellular alkalization. NHE1, V‐ATPase and NBCn1 activities associate with cancer malignity. From data in 16, 22, 27, 28, 29, 30, 33, 75.

The cell metabolism results in the continuous generation of acid‐base equivalent species, including protons (H^+^) (end product of the glycolytic metabolism), lactate (generated under low O_2_) and carbon dioxide (CO_2_) (derived from mitochondrial‐dependent macromolecules metabolism) 17. Mammalian cells express different families of membrane transporters that control the content of acid‐base equivalents (Fig. 1). These transport systems modulate pHi by exporting intracellular acid equivalents to the extracellular space or endosomes, by incorporating equivalents of bases, or both 18, 19. Under physiological conditions H^+^ are exported by sodium (Na^+^)/H^+^ exchangers (NHEs) 18, vacuolar H^+^ ATPases (V‐ATPase) 19 and H^+^/potassium (K^+^) ATPases 17. The Na^+^/HCO_3_^−^ transporters (NBCs) regulate both Na^+^ and HCO_3_^−^ 20, and the bidirectional monocarboxylate transporters (MCTs) regulate the intracellular content of the conjugate base lactate 21. However, in several diseases, including cancer, alterations in the pH value and the mechanisms involved in the reversion to a physiological pH are reported 22, 23, 24. Only NHEs 18, 22, 23, 25, 26, NBCs 27, 28, 29, 30 and V‐ATPases 31, 32, 33 have been documented to potentially play a role in human gynaecological cancers. Thus, abnormal function of these membrane transport mechanisms controlling the pHi and pHo may have harmful consequences for cell function.

NHEs belong to a family of electroneutral membrane transporters removing H^+^ to the extracellular media or intracellular organelle against a H^+^ electrochemical gradient in exchange with Na^+^ 34. This phenomenon increases pHi (alkalization) and decreases pHo (acidification). NHEs family includes 11 members of which the NHE1 isoform is the most characterized because of its role in cancer 22, 25, 26. NHE1 expression is increased during tumour malignant transformation 18, 23, suggesting a role for NHEs, particularly NHE1, in cancer.

Membrane transporters that carry bicarbonate (HCO_3_^−^) play important roles in regulating both pHi and pHo 20. In mammals, the primary mechanisms accounting for HCO_3_^−^ transport include at least three functional membrane transporters, *i.e*. NBCs, the Cl^−^/HCO_3_^−^ (AEs) and the Na^+^‐driven Cl^−^/HCO_3_^−^ (NDCBEs) electroneutral exchangers. Functional studies demonstrate that NBCs may remove intracellular HCO_3_^−^ to the extracellular media leading to intracellular acidification or take up HCO_3_^−^ leading to intracellular alkalization 35. NBCs include the electroneutral cotransporters (NBCn) that cotransport one molecule of Na^+^ and one molecule of HCO_3_^−^ and the electrogenic cotransporters that cotransport one molecule of Na^+^ and two or three molecules of HCO_3_^−^ 27, 36, 37, 38. One of the roles of AE1 isoform in gastric cancer regards with induction of cancer progression 39 and cancer cell proliferation 40, while AE1 knockdown suppresses tumour growth *in vivo* 40. Whether a reduced removal and increased take up of HCO_3_^−^ *via* these mechanisms is responsible of the intracellular alkalization in epithelial ovarian cancer is unknown 20, 22, 41, 42.

V‐ATPases contain a cytosolic V1 domain, responsible for ATP hydrolysis, and an integral V_0_ domain, responsible for H^+^ translocation 43, 44. Modulation of V‐ATPase expression and activity results in pHi control. To date, fusion of V‐ATPase‐containing exocytic vesicles with the plasma membrane leads to increased H^+^ transport, but increased V‐ATPases endocytosis results in lower H^+^ transport into the extracellular medium 45, 46. In a recent study, it was shown that V‐ATPase‐V0α2 isoform is apparently increased in ovarian tumour cell surface 47. Thus, V‐ATPases are considered as possible targets for cancer therapy, including ovarian cancer, because of its role in anoikis resistance, metastasis and cancer cell invasion 31, 32, 33, 47.

## Functional link between nitric oxide and pH in gynaecological cancers

### Nitric oxide in gynaecological cancers

Patients with cancer show altered NOS expression and activity. To date, the expression of iNOS is higher and correlates with poor prognosis in breast cancer 48. Exposure of cancer cells to high levels of nitric oxide results in a more aggressive phenotype in breast cancer cell lines, enhancing its resistance to apoptosis. An excellent discussion on nitric oxide roles in human ovarian cancer was recently summarized 49. It is clear that there is conflicting data in the literature in terms of iNOS expression as a prognostic factor. A low nitric oxide level is shown to trigger pro‐proliferative/pro‐survival responses characterized by the induction of antiapoptotic genes and angiogenic factors, but a high nitric oxide level triggers pro‐apoptotic/cytotoxic responses. The pro‐proliferative/pro‐survival function of nitric oxide is mediated by cGMP, but the pro‐apoptotic/cytotoxic function seems related to be independent of nitric oxide/cGMP signalling pathway but to the generation of reactive‐nitrogen species in ovarian cancer cells. Recent studies show increased angiogenesis in ovarian cancer due to the overexpression of the *DLX4* homeobox gene 50 and cancer growth in ovarian high‐grade serous carcinoma due to reduced expression of PDZ‐LIM domain‐containing protein 2 (a cancer growth repressor) 51 following increased iNOS‐derived nitric oxide. However, limited information is available about the functional role of nitric oxide in invasion in gynaecological cancer. It was shown that the nitric oxide donor spermine and diethylenetriamine reduce transmigration of human ovarian cancer cell lines OVCAR3 and SKOV3 and the matrix metalloproteinase 2 activity in SKOV3 cells, thus reducing their metastatic potential 52.

**Figure 2:**
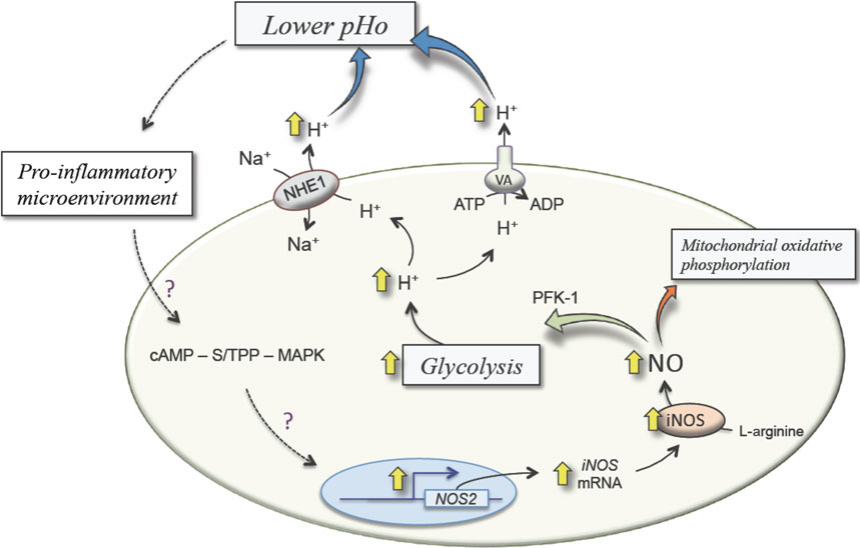
Potential involvement of nitric oxide in the extracellular medium acidification in cancer. In cancer cells, the inducible nitric oxide synthase (iNOS) is overexpressed (⇧) due to a higher *iNOS* mRNA expression as a result of increased transcription of *NOS2* gene (*NOS2*). Increased iNOS expression results in conversion of L‐arginine (L‐Arginine) into L‐citrulline and nitric oxide. The nitric oxide reduces (red arrow) mitochondrial oxidative phosphorylation at complex III and complex IV of the electron transport chain (*Mitochondrial oxidative phosphorylation*), but increases the activity of the 6‐phosphofructokinase 1 (PFK‐1) resulting in the activation (green arrow) of glycolysis (*Glycolysis*) promoting a metabolic switch from an aerobic to a glycolytic phenotype (Warburg effect). Glycolysis promotes H^+^ generation, which is removed from the intracellular to the extracellular space in exchange with sodium (Na^+^) *via* the Na^+^, proton (H^+^) exchanger isoform 1 (NHE1) and through the plasma membrane located vacuolar H^+^‐ATPases (VA) at expenses of ATP hydrolysis into ADP. These membrane transport mechanisms contribute (blue arrows) to the acidification of the extracellular space (*lower pHo*). Acidification of the extracellular space promotes a pro‐inflammatory microenvironment (*pro‐inflammatory microenvironment*) that is likely (?) to activate signalling pathways involving cyclic AMP – serine/threonine protein phosphatase – mitogen‐activated protein kinases (cAMP – S/TPP – MAPK). Additionally, an induction of *NOS2* expression by not yet defined mechanisms is likely (?). Thus, a potential positive feedback loop or ‘*vicious cycle*’ maintained by the extracellular acidification and iNOS induction in proposed to occur in cancer. From data in 14, 16, 22, 33, 48, 53, 54, 61, 62, 85.

Nitric oxide also modulates epithelial ovarian cancer cells metabolism, promoting cancer cell growth and inhibiting mitochondrial oxidative phosphorylation 14. Thus, nitric oxide plays a role in developing a glycolytic phenotype (*i.e*. Warburg effect) and reducing mitochondrial respiration in an anaerobic environment leading to increased lactate generation and acid secretion to the extracellular medium 53, 54. Paradoxically, increased intracellular generation of lactate could instead result in decreased pHi. One possible explanation for this phenomenon is that following lactate generation, it is removed to the extracellular media by MCTs 21, thus increasing pHi and reducing pHo. As MCT1 and MCT4 forms are expressed at the plasma membrane in ovarian cancer tissues 55, this phenomenon is a possibility. In addition, preliminary observations suggest that NHE1 could also be expressed in ovarian cancer tissue 16. Thus, a coordinated activity between MCTs and NHE1 could lead to intracellular alkalization and extracellular acidification in ovarian cancer. Alternatively, HCO_3_^−^ influx could contribute to intracellular alkalization. Interestingly, the mRNA expression of NDCBEs was detected in human ovary 56; however, additional studies are required to address the contribution of NDCBEs to the pHi control in human ovarian cancer. Therefore, nitric oxide is involved in pH modulation in cancer cells contributing to an acidic extracellular microenvironment (Fig. 2). This phenomenon results in degradation of the extracellular matrix, promoting a prometastatic cellular behaviour 57, 58. Thus, it is feasible that nitric oxide may play a role in metastasis and cancer cell migration, lowering the pHo in gynaecological cancers 59, 60. Interestingly, targeting nitric oxide synthesis is proposed as a potential therapy for treatment in patients with ovarian cancer 48, 60. It was reported that a low pHo interfered with signalling pathways that induce iNOS expression in L929 fibrosarcoma cell lines 61. Therefore, reducing iNOS expression may influence tumour progression 61. Nevertheless, the potential interdependence of a low pHo and iNOS regulation in cancer opens the possibility that a reduced pHo may have a consequence on iNOS expression and activity in human ovarian cancer.

Expression of iNOS was detected in 50% of primary tumours and 62% of metastatic lesions in human epithelial ovarian cancer 4. However, iNOS expression is blunted in benign lesions, but positive in 60–70% of *in situ* breast carcinomas 62. Interestingly, high iNOS expression results in worse survival compared with patients with low iNOS expression. Additionally, a correlation between a low risk of disease recurrence and higher iNOS expression was reported in tumour cells from human uterine cervix carcinoma 63. In human epithelial ovarian cancer, the use of cisplatin, an alkylating‐like agent used in ovarian cancer chemotherapy 3, results in iNOS up‐regulation, but eNOS down‐regulation of expression, suggesting a pivotal role of iNOS in this disease 5. Other studies show that eNOS overexpression in endometrial cancer correlates with lower disease‐free patient survival, and increased myometrium invasion with increased cytoplasmic eNOS expression 64. Interestingly, both the *NOS3* polymorphism G894T and variable number tandem repeats polymorphism in the intron 4 are proposed as factors of susceptibility for endometrial cancer associated with increased nitric oxide synthesis 65. As E298D and −786T>C polymorphisms in *NOS3* result with lower eNOS expression and nitric oxide synthesis and reduced risk for breast cancer 66, eNOS, as well as iNOS, may play roles in the aetiology of gynaecological cancers. Furthermore, the modulation of the activity of these enzymes may have a beneficial impact on reducing human gynaecological cancer malignancy.

### pH in gynaecological cancers

Excessive production of H^+^ and changes in pHo/pHi is a condition where cancer cells must adapt to proliferate and migrate 23, 67, 68, 69. In cells from normal tissues, the pH is maintained at physiological values reaching pHi ~7.0 and pHo ~7.4. However, these values are changed in cancer cells, with pHi ~7.7 and pHo ~6.5 26, 34, a phenomenon seen in human solid tumours 70 that result in higher cancer aggressiveness, invasion and cell metabolism. Extracellular acidification induces changes in lysosomes trafficking, allowing the release of lysosomal content to the extracellular media 71. Lysosomes contain several proteases, including cathepsin B, an extracellular matrix degradation enzyme that degrades the extracellular matrix 72, 73. Thus, the release of lysosomal content into the extracellular medium by a change in the pHi/pHo will increases tumour cell invasion 71. Overexpression of NHE1 is reported to associate with higher cell migration in cervical cancer cells 74. Additionally, NHE1‐increased expression correlates with lower disease‐free survival probability and overall survival in patients with this disease 74. These findings suggest that NHE1 has a potential prognostic factor in these patients. However, even when preliminary results suggest that NHE1 is expressed in biopsies from human ovarian cancer, ascites‐tumour cells and ovarian cancer cell lines 16, there are no reports addressing the regulatory mechanism(s) involved in both the NHE1 expression and activity in this type of cancer. However, the role for NHEs in other types of cancer has been addressed. To date, the expression of NHE1 28, 75, 76 and NHE7 77 enhances cell proliferation, invasiveness and metastasis in breast cancer cells. Other studies show that NBCs are expressed in Chinese hamster ovary cell lines, as well as in breast, colorectal, pancreatic and cervical cancer and sarcoma 29, 30. Thus, the potential role of the pH as a modulator of these types of cotransporter is expected from studies in breast cancer. In the MDA‐MB‐435 and MDA‐MB‐231 breast cancer cell lines, NBCn1 activity sustains an alkaline pHi and acidic pHo, both conditions increasing cell proliferation rate and cell migration and invasion 28. Also, NBCn1 expression is higher in low‐grade lesions compared with high‐grade lesions or healthy tissue in grades II–III breast cancer. Thus, NBCn1 was suggested to play a role in cancer progression 27, 28, 38. Interestingly, by assaying the pHi recovery rate after an NH_4_Cl pulse in freshly isolated tissue from patients with grades I, II and III breast cancer, a similar functional involvement was estimated for NBCs and NHE1 27. NBCs seem to be more effective controlling the pHi in a range close to physiological values compared with NHE1 that is more effective controlling the pHi at a more acidic intracellular value 27. Even when these findings suggest that extracellular acidification affects the membrane transporters controlling pHi, and that this phenomenon could be differential for NBCs and NHE1, more information is needed to understand the effect of an acidic extracellular microenvironment and pHi control systems in human ovarian cancer.

### Functional link between nitric oxide and pH in gynaecological cancer

As several pathologies course with altered pH and nitric oxide generation, it is plausible to consider that pH alteration may affect nitric oxide signalling impacting a wide variety of physiological/pathological processes. A change in the pH alters the vascular tone leading to either vasodilation or vasoconstriction 78. As vascular cells are highly permeable to H^+^, such as the arterial vascular smooth muscle cells, changes in pHo could cause changes in pHi 78. The increase in intracellular H^+^ content affects Ca^2+^ signalling and nitric oxide synthesis in HUVECs and in rat coronary endothelial cells 79, 80, 81. However, this apparent dependency of a change in pHi due to a change in the pHo is not a generalized phenomenon. To date, the exposure of rat aorta endothelial cells to an alkaline medium (pH ∼8.5) results in higher pHi and intracellular nitric oxide level 82. However, exposure to an acidic extracellular medium (pH ∼6.5) had no effect on pHi, but increased the intracellular nitric oxide level. Thus, under a low pHo, the nitric oxide synthesis is increased by a mechanism other than intracellular medium acidification in this cell type. Additionally, incubation of rat aortas in a medium at pHo ∼7.0 lowers the pHi only in the smooth muscle layer 82. This finding suggests that vascular endothelium and smooth muscle cells have differential mechanisms to control the pHi in response to extracellular acidification.

The altered pH value defines a common characteristic of malignant cells 83, 84. Extracellular acidification facilitates tumour invasion and reduces immunosurveillance, thus contributing to cancer malignity 23. An acidic microenvironment (pH <7.2) reduces nitric oxide synthesis, increases cell viability and blocks iNOS induction in the L929 mouse cancer fibroblast cell line 61. Thus, acidic microenvironments result in down‐regulation of iNOS‐dependent synthesis of nitric oxide in tumour cells. However, fibroblasts from normal rats exposed to an acidic microenvironment (pHo ~6.6) show decreased pHi (~6.85–6.50), leading to a proinflammatory response characterized by induction of iNOS as well as tumour necrosis factor α, and cyclooxygenase 2 85. Under this condition, nitrite and nitrate generation was higher compared with a non‐acidic microenvironment. It was suggested that intracellular acidity results in inflammation leading to iNOS induction and increased nitric oxide level in non‐tumour cells. These findings are certainly intriguing and suggest that acidic microenvironments may result in either down‐regulation in tumour cells or up‐regulation of iNOS‐dependent synthesis of nitric oxide in non‐tumour cells.

## Conclusion

In human diseases, both the NOS expression and activity and the pH are altered, affecting a variety of processes including tumour cells metabolism. Also, expression and activity of H^+^ exchangers (mainly NHE1) are increased in tumour cells. We proposed a sequence of events that may link intracellular events (glycolysis, acidic pHi, NOS2 expression) with extracellular medium (alkaline pHo, generation of a proinflammatory microenvironment), *i.e*. a potential functional link, leading to higher nitric oxide generation in human epithelial ovarian cancer cells (Fig. 2). NHEs and other membrane transporters lead to H^+^ efflux from the cytoplasm to the extracellular media, resulting in the generation of an acidic extracellular microenvironment. This condition also generates a pro‐inflammatory medium inducing iNOS expression and nitric oxide synthesis. Furthermore, increased nitric oxide bioavailability inhibits mitochondrial respiration, resulting in higher H^+^ generation due to increased glycolytic metabolism. Based on the available information, it is suggested that targeting NHEs and iNOS may be an option to alter a potential tumour microenvironment vicious cycle in human epithelial ovarian cancer. Unfortunately, there are no studies addressing whether increased nitric oxide will result in higher NHEs‐dependent cell growth or invasiveness of human ovarian cancer cells (see reviews 49, 86). However, it is necessary to keep in mind that most of the available information supporting this proposal was concluded from *in vitro* assays. Thus, realistic conditions in malignant diseases, especially in human gynaecologic cancer, are still in doubt.

There are no reports addressing a potential regulation of the pHi or pHo by nitric oxide in cells from epithelial ovarian cancer 22. Indeed, a role for nitric oxide as a modulator of NHEs, including NHE1, even in normal tissues, is rather controversial. As an example, in rat isolated ventricular myocytes, exogenous nitric oxide reduced the pHi increasing NHEs activity 87. This finding contrasts with an earlier study showing that exogenous nitric oxide caused a cGMP‐dependent decrease in NHEs activity in this cell type 88. None of these studies showed whether there is a correlation between the pHi changes and NHEs isoform expression. Thus, it is not possible to answer the question whether nitric oxide causes this effect associated to a change in NHE1 and other NHEs isoform's protein expression. Interestingly, a reduced activity of NHE4, but not NHE1 or NHE2 isoforms, without altering its protein expression level, was recently suggested in T_84_ cells (from human colon carcinoma) in response to *Escherichia coli* strains‐released heat‐stable enterotoxins 89. The potential mechanism explaining this phenomenon does not involve a response to nitric oxide or cGMP, but to cAMP 89. Thus, not only NHE1 but also other membrane transport mechanisms may play a role in this phenomenon. It seems urgent to unveil mechanisms associated with pHi and pHo modulation by nitric oxide in either normal or cancer cells. As this information is unknown in human ovarian cancer cells, including epithelial ovarian cancer 3, only speculations are for now available. Interestingly, in a single case report a patient with metastatic renal cancer treated with NaHCO_3_, there was a lower severity of the associated liver lesions 90. This phenomenon was proposed to result from a peritumoral alkalization, leading to reduced tumour growth and invasion. Thus, translational studies are required to value these types of approaches as a potential clinical therapy in human ovarian and other types of cancer.

## Conflict of interest

The authors confirm that there are no conflicts of interest.

## Author contribution

Designed research study (CS, AL, DIC, FP, JG, LS), collected clinical data (AL, FP, EB), collected and analysed literature information (CS, JA, LN, EB, MS, FT, JG, DIC, FP, AL, LS), designed the figures (CS, JA, LS) and wrote the article (CS, JA, LN, EB, MS, LS).
